# “Indigenous models of social prescribing”–a six-domain framework for social wellbeing and other support services delivered by Aboriginal Community Controlled Health Organisations

**DOI:** 10.1016/j.lanwpc.2026.101999

**Published:** 2026-10-16

**Authors:** Uday Narayan Yadav, Chelsea Liu, Reece Griffin, Rosemary Wyber, Isabel Hanson, Odette Pearson

**Affiliations:** a National Centre for Epidemiology and Population Health, The Australian National University, Canberra, ACT, Australia; b International Centre for Future Health Systems, University of New South Wales, Sydney, Australia; c The Poche Centre for Indigenous Health, Faculty of Medicine and Health, The University of Sydney, Sydney, Australia; d Yardhura Walani, National Centre for Aboriginal and Torres Strait Islander Wellbeing Research, The Australian National University, Canberra, ACT, Australia; e Torres and Cape Hospital Health Service, Queensland, Australia; f The Kids Research Institute Australia, Perth, Western Australia; g Nuffield Department of Primary Care Sciences, University of Oxford, Oxford, United Kingdom; h South Australian Health and Medical Research Institute, Adelaide, South Australia, Australia; i Faculty of Health and Medical Science, Adelaide University, Adelaide, South Australia, Australia

**Keywords:** Aboriginal community-controlled health organisations, Framework, Holistic model of care, Social and cultural determinants

## Abstract

This viewpoint examines the literature to illuminate the role of Aboriginal community-controlled health organisations in supporting access to social wellbeing and other support services that address the social and cultural determinants of health and wellbeing for Aboriginal and Torres Strait Islander peoples. Our papers call on primary care providers, researchers, and policymakers to apply and test the six-domain framework we developed to describe their engagement with social wellbeing and other support services, and to showcase their work, control, and financial needs.

## Background

Aboriginal and Torres Strait Islander people view health beyond physical well-being to encompass ‘*the social, emotional, spiritual, and cultural well-being for both individual and whole Community’*.^1^ This community-led holistic definition of health powerfully recognises the determinants of health and wellbeing outcomes. In Australia, 35% of the health gap between Aboriginal and Torres Strait Islander communities and non-Indigenous Australians is attributed to social determinants. Another 35% of the gap remains unexplained, potentially including cultural determinants not captured in the Australian Bureau of Statistics health survey.^2^^,^^3^

Following the invasion and colonisation of Australia, Aboriginal and Torres Strait Islander people have experienced persistent political, health, economic, and social inequalities. These inequalities are due to structural disadvantage, including systematic discrimination, racism, and ongoing movements of resistance groups targeting Indigenous perspectives, beliefs, and practices.^4^ Despite ongoing inequities, Aboriginal and Torres Strait Islander peoples continue to demonstrate strengths, tenacity and resilience, drawing on social, cultural and economic protective factors that promote community health and wellbeing.

Health services serving Aboriginal and Torres Strait Islander peoples have a clear mandate to respond to social and cultural needs.^5^ Holistic, comprehensive, culturally safe primary care, including attention to social, cultural and biomedical determinants of health and wellbeing has been central to Aboriginal community-controlled health organisations (ACCHOs) in Australia since 1971.^6^ In 2019, the National Aboriginal Community Controlled Health Organisation (NACCHO) enshrined this within the ACCHO Core Services and Outcomes Framework, formalising a sector-wide commitment to ‘community health promotion and empowerment.^7^ This holistic model of culturally safe care contributes to addressing the social and cultural determinants that shape health and wellbeing outcomes.

Internationally and within non-Indigenous Australian contexts, linking patients to social wellbeing and other support services — such as housing, income insurance, employment, physical activity groups, art therapy, meditation, community gardens, arts and music — has been referred to as ‘social prescribing’ or ‘social referral initiatives’.^8^ These kinds of connections to social wellbeing and other support services are already commonplace within ACCHOs' holistic model of care, but are rarely labelled as ‘social prescribing’ or ‘social referral’.^9^ A recent feasibility study on Social Prescribing in the Australian Context advocates for learning from and supporting the integrated models of care pioneered by ACCHOs.^10^ Ensuring that the leadership and experiences of ACCHOs in this space are appropriately recognised and valued requires frameworks that describe how ACCHOs connect patients to a range of health, social wellbeing, and other support services.

This article explores different mechanisms of ACCHOs supporting access to services for the social and cultural determinants of health and wellbeing for Aboriginal and Torres Strait Islander peoples. It considers a targeted, concept-driven critical review that typically results in the development of a framework.^11^ The search was conducted across Google Scholar, PubMed, and publicly available ACCHO and NACCHO publications to identify publications that have explicitly considered Aboriginal and Torres Strait Islander communities in relation to social and cultural determinants of health. In particular, describing this spectrum of ACCHOs, we explore opportunities for these efforts to be more widely recognised as a model within Australia's social prescribing landscape.

## ACCHOs as a ‘one-stop shop’ for patients, addressing social and cultural determinants of health and wellbeing

ACCHOs commonly function as a ‘hub’ or a ‘one-stop shop’ for patients to access or be connected to a broad range of supports, spanning health care, social wellbeing and other support services. This integrated approach can improve access and equity across social, educational, economic and health systems. Evidence shows that ACCHOs are pivotal in community empowerment and promoting positive health determinants by acknowledging Aboriginal health concepts and providing culturally safe care.^6^

ACCHOs have a foundational role in addressing social determinants through employment, training and workforce development. They are the largest employer of Aboriginal and Torres Strait Islander people in Australia, employing over 3500 Aboriginal people, who comprise 55% of the total ACCHOs workforce.^12^ The National Aboriginal and Torres Strait Islander Health Workforce Strategic Framework and Implementation Plan (2021–2031) noted that ACCHOs are the primary setting for training Aboriginal and Torres Strait Islander people in Aboriginal Health Worker positions, including through in-service traineeship models and pathways to tertiary education and professional training in culturally appropriate health care.^13^

ACCHO activities also extend across a wide range of social wellbeing and support services, including housing support, art therapy,^14^ education,^15^ transport,^16^^,^^17^ accommodations for homeless people,^6^^,^^18^ justice-related services,^6^ informal childcare, nutrition programs, and behavioural health supports, such as physical activity programs and drug, alcohol and substance misuse services.^19^^,^^20^ The published literature^6^^,^^19^ also outlines activities as part of ACCHOs' comprehensive primary health care model. In some settings, particularly remote areas, they provide breakfast programs, elders’ lunches, aged care facilities, and assistance for the homeless. They also contribute to disaster preparedness and response,^21^ including during bushfires, floods, COVID-19 pandemics and cyclones. Many ACCHOs maintain strong links with legal and justice systems, including through co-located services in clinics, communities or prisons, and through post-release support for incarcerated people and their families.^6^^,^^22^^,^^23^ In addition, some ACCHOs provide or facilitate access to traditional healing services, smoking cessation clinics, parental support programs, and broader health and wellbeing promotion activities.^6^^,^^16^^,^^20^

ACCHOs also contribute to community development by conducting or referring to activities beyond health, such as cultural days and camps, support for local sporting teams, reconciliation events, and initiatives that foster social connectedness.^16^^,^^19^^,^^20^ ACCHOs also play a leading role in providing culturally appropriate health information through health promotion resources, social media,^23^ and informal conversations.^16^^,^^24^

## Spectrum of social wellbeing and other support services delivered *by ACCHOs*

The scope of activities delivered or supported by ACCHOs is extensive and encompasses a broad range of social wellbeing and other support services. Using a conventional content analysis approach,^25^ we systematically coded ACCHO service functions related to social wellbeing and other support services. This analysis identified distinct meaningful categories of ACCHO involvement across diverse programs and activities that subsequently formed the basis of the six-domain framework.

We explored the resonance of the proposed six-domain framework with two ACCHOs and a NAACHO representative during the development process. The framework was presented at the 2025 Lowitja Institute's 4th International Indigenous Health and Wellbeing Conference^26^ to gather sector feedback from researchers, practitioners, and community-controlled sector stakeholders. As illustrated in Fig. 1, increasing levels of ACCHO involvement (vertical axis) correspond to the degree of control (horizontal axis), as well as greater oversight and Aboriginal and Torres Strait Islander cultural input in program and service delivery.

**Fig. 1:**
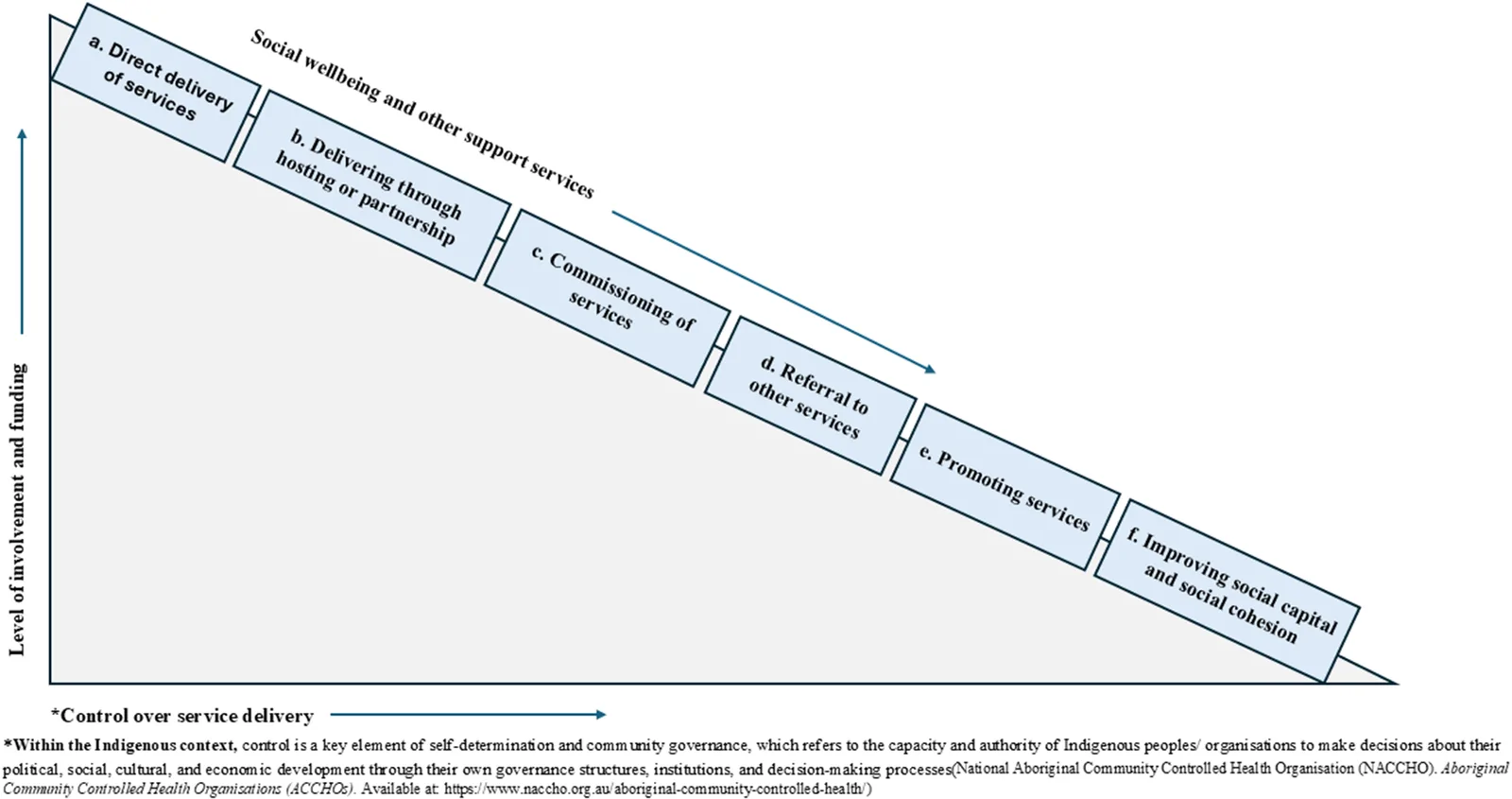
*Spectrum of social well-being and other support services delivered by ACCHOs*.

### *Operational definition of the six domains of* social wellbeing and other support services delivery *by ACCHOs*

a) **Direct delivery of services:** Delivery of funded services or initiatives directly by the ACCHO to individuals. It involves the direct consumption of ACCHO resources to deliver the services. E.g., Tackling Indigenous Smoking and Social and Emotional Wellbeing Programs.
b) **Delivery through hosting or partnerships:** ACCHOs deliver services to people indirectly through partnerships with service providers or co-location of other service providers in the ACCHO space. E.g. National Disability Insurance Scheme, Aged Care, health justice, food insecurity.
c) **Commissioning of services:** Through commissioning, ACCHOs allocate funding and contract with other organisations to provide services that they cannot provide directly. This may include allied health services,^27^ training for clinicians and staff,^6^ or research contracts for needs assessment or evaluation,^28^^,^^29^ As a funder, ACCHOs may have financial and contractual control but less day-to-day operational involvement.
d) **Referral to other services:** Arranging services by navigating or connecting people to other service providers or organisations through a formal referral process to meet their needs. E.g., Rangers program, Homelessness support, Men and Women Shed program and walking groups
e) **Promoting services:** Passively providing information about other services through social media or pamphlets, etc., where ACCHOs do not partner, provide referrals, or contact service providers directly. E.g., Information sessions and posters
f) **Improving social capital and social cohesion:** Activities related to employment creation, capacity-building training opportunities to obtain qualifications,^12^ supporting families in educating their children, community empowerment initiatives, cultural immersion workshops and advocacy, including reducing alcohol-related harm in Aboriginal communities.^6^ E.g. employment initiatives and community empowerment

The range of services ACCHOs provides varies across settings and is tailored to each community's needs. These services may span multiple domains across the spectrum of social wellbeing and other supports, including healthy lifestyle sessions and information sessions (e.g., smoking cessation, physical activity), diabetes and heart education, and drug and alcohol services. ACCHO partners may include the Department of Communities and Justice, Drug, Alcohol and Addiction Services, Legal and Justice Services, Family Services, interpreter services, and non-governmental service providers.^30^ Table 1 presents examples of initiatives across the ACCHO sector that address social and cultural determinants of health and wellbeing.^30^, ^31^, ^32^, ^33^, ^34^, ^35^, ^36^, ^37^

**Table 1:** ACCHO sector initiatives (not an exhaustive list of initiatives) for addressing social and cultural determinants of health and wellbeing.

Name of the programs/initiatives	Description	ACCHO role
Social and emotional wellbeing programs	SEWB programs focus on Cultural, Social and Emotional well-being (such as connection to country, culture, family, community and spirit) with an effort to reduce psychological distress, self-harm and suicide among Aboriginal and Torres Strait Islander communities. For example, in Perth, A social and well-being team uses yarning style conversations to assess holistic needs and provide a range of services, such as cultural connection by supporting travel to the country, engagement with family members, engagement in cooking or relevant hobbies groups and making new friends.^31^ In Far North Queensland, individuals were also invited to participate in various programs depending on their needs, such as the Tackling Indigenous Smoking program, a needle exchange program, a youth diversion program, a home maintenance program, a parenting group, men's and women's groups, and healthy cooking groups.^30^ SWEB models adopt a strengths-based approach informed by local needs and guiding principles to address the heterogeneous cultural needs of Aboriginal and Torres Strait Islander peoples in Australia.	Direct delivery of services
Health justice partnerships	Some ACCHOs partner with legal service providers (community legal centres or legal aid commissions) to provide on-site legal services to the people. Legal professionals provide accessible legal support to individuals experiencing complex needs, such as credit and debt, fines and payday loans, Centrelink, housing issues, family breakdown, family violence, child protection and minor crime, including addressing systemic factors that contribute to social and health inequity.^32^	Delivery through hosting or partnerships
ACCHO aged care service provision	Some ACCHOs support Elders in navigating internal and external services to meet their needs, including residential aged care services, home care services and day respite care.^33^ ACCHOs establish referral clinics that support referrals from their health and community programs to Aged Care service providers who do assessments for Elders for a range of home care services such as cleaning, lawn mowing, home modifications, transport services, showering, dressing and mobility support.^34^	Referring or connecting
Aboriginal quitline	Clinical support for smoking cessation is delivered directly through ACCHO clinical services and also through connections to other services. For example, many ACCHOs display posters or social media information about how to contact the Aboriginal Quitline for additional support.^35^	Promoting services
Other initiatives by ACCHOs	The Kimberley Aboriginal Medical Services established the Solid Connections program to help Aboriginal and Torres Strait Islander people discover new skills and career pathways.^36^ ACCHOs also play a key role in championing alcohol-related harm reduction in local Aboriginal communities.^37^	Improving social capital and social cohesion

## Implications of the six-domain framework for ACCHOs

The NACCHO Core Services and Outcomes Framework makes clear that the remit of ACCHOs extends beyond clinical care.^7^ The delivery and support of these social wellbeing and other support services occur across a spectrum of engagement and control. Describing this spectrum has several benefits.

First, it enables ACCHOs to clearly articulate their level of input to community members and governance bodies, including the extent to which they have control or influence over particular services. This transparency is important for accountability, particularly where externally provided services vary in cultural safety, and can highlight the absence of community-controlled providers in settings where they are needed.^30^ It can also support ACCHOS to identify service gaps, advocate for appropriate funding, and strengthen community control in areas where cultural safety and governance are essential.

Second, a structured framework can help make visible some of the often-unrecognised work undertaken by ACCHOs by mapping how they contribute to the delivery and success of programs provided by other organisations. For example, when ACCHOs host in-reach services or display promotional materials, they may provide implicit endorsement of those services through their trusted relationships with the community. This creates cultural and reputational responsibilities for ACCHOs. In practice, hosting in-reach services may also involve practical support, such as reception staff responding to questions, providing directions, or receiving feedback about service experience. ACCHOs’ role as cultural knowledge and logistic brokers in these settings is often unrecognised and frequently unfunded.^24^^,^^38^

Third, a systemic approach to defining roles may support inter-agency collaboration by establishing shared goals, values, language and understanding of partnership. While collaboration between ACCHOs and partner organisations can contribute to system reform and improve outcomes for Aboriginal and Torres Strait Islander communities, partnerships are often challenged by complex and unstable service networks, insecure funding mechanisms, and limited accountability for the cultural safety of partner-delivered services. Recent evidence from Far North Queensland shows that over 80% of upstream social, emotional and wellbeing services were delivered by non-governmental organisations.^30^ This raises important questions about Aboriginal and Torres Strait Islander participation in decision-making, control over service delivery, and securing funding to strengthen services provided by ACCHOs to address social and cultural determinants.^33^ It also highlights the need to consider the extent to which non-Indigenous organisations can deliver culturally safe services.

While this paper highlights the role of ACCHOs in delivering and supporting social wellbeing and other support services, several challenges and considerations must be recognised. ACCHOs are often expected to support a broad range of activities despite already stretched organisational resources, and this work is frequently not recognised in funding models that prioritise clinical key performance indicators over holistic, community-identified needs. Where ACCHOs hold advisory rather than controlling roles, their capacity to influence service quality through cultural standards may be limited, posing reputational risks. In such contexts, clear governance and escalation pathways are needed when external service providers do not meet agreed standards.

Funding structures, commissioning arrangements, and workforce shortages may limit ACCHOs' ability to expand their full remit, even when community demand is strong. These can be addressed by coordinated action across policy, commissioning and workforce bodies rather than relying solely on ACCHOs to compensate for structural limitations. Importantly, a national funding model is required to adequately support the ACCHO sector to provide flexible, holistic services to address the unmet needs of Aboriginal and Torres Strait Islander peoples.^9^

## Conclusion

ACCHOs are intentionally and intimately connected to the needs of communities. The sector has a broad remit to directly deliver, host, commission, connect, promote, and improve social capital and cohesion. Describing the network of partnerships that operationalise these relationships can be challenging. This proposed six-domain framework offers an opportunity for care providers, researchers, and policymakers to test this approach and expand our foundational consultations with ACCHOs and other sectors. Importantly, we highlight the need for national-level research to understand and showcase best-practice initiatives, control, and financial needs in ACCHOs that address social and cultural determinants. Insights from such evaluations will guide the implementation and evaluation of programs that address the broader determinants of people's health in a broader Australian context.

## Contributors

UNY: led idea conceptualisation, led the literature synthesis and critical analysis, writing–original draft, visualisation, writing–reviewing and editing; CL: writing–reviewing, editing and referencing; RC and IH: writing–reviewing and editing; RW: conceptualisation, writing–reviewing and editing; OP: supervision, writing–reviewing and editing.

## Authors' information

Uday N Yadav (Madhesi, Nepal), Odette Pearson (Kuku Yalnji/Torres Strait Islander) and Reece Griffin (Awabakal).

## Declaration of interests

UNY, RW and OP are recipients of National Health and Medical Research Council (NHMRC) research funding. All authors declare no competing interests. The authors declare that the present research was conducted in the absence of any commercial or financial relationships that could be construed as potential conflicts of interest.
